# Validation of the Polar OH1 and M600 optical heart rate sensors during front crawl swim training

**DOI:** 10.1371/journal.pone.0231522

**Published:** 2020-04-16

**Authors:** Bjørn Harald Olstad, Christoph Zinner

**Affiliations:** 1 Institute of Physical Performance, Norwegian School of Sport Sciences, Oslo, Norway; 2 Department of Sport, University of Applied Sciences for Police and Administration of Hesse, Wiesbaden, Germany; São Paulo State University (UNESP), BRAZIL

## Abstract

**Purpose:**

The Polar OH1 is an optical heart rate (HR) sensor which can be used on different parts of the body. The purpose of the study was to evaluate the validity of the OH1 as well as a wrist worn heart rate device (Polar M600) during swimming.

**Methods:**

Twenty-six well-trained competitive swimmers performed a regular training session including different swimming intensities. During the training the swimmers wore a H10 HR sensor with Polar Pro strap (H10) underneath the swim suit, a Polar OH1 optical HR sensor (OH1) underneath the swimming cap at the temple, and a sports watch with optical HR sensor, Polar M600 smart watch (M600) on the wrist.

**Results:**

No difference in HRmax, HRmean and HRmin between H10 and OH1 were evident. The HRmax and HRmean obtained by the M600 was significantly lower than the obtained by H10 and OH1 (p < 0.05). The ICC showed mostly excellent agreements between H10 and OH1 and poor to good agreements between H10 and M600. Bland-Altmann plot for M600 vs. H10 indicates upper and lower limits of agreement of -53.0 to 33.9 beats per minute. For OH1 vs. H10 the upper and lower limits of agreement were -26.9 to 24.7 beats per minute.

**Conclusion:**

The Polar OH1 optical HR sensor is a valid tool to monitor HR of different intensities during swimming whereas the Polar M600 smart watch as a wrist worn device is less accurate.

## Introduction

Swimming is a sport with a high overall training load throughout the year [1]. To improve performance carefully planned modifications in training load are required, particularly increases in frequency, duration, and intensity. Monitoring the training load of a swimmer with high amounts of training is important to i) control whether he or she is adapting positively to the training and ii) to minimize the risk of illness, non-functional overreaching or even overtraining [2].

During swimming the external load is defined as the distance covered in pre-defined velocities [3]. To get an approximation of the internal load several markers are available for coaches, athletes, and scientists [2]. However, very few of these markers have strong scientific evidence and/or their usage is not reasonable from an ecological point of view. In this context heart rate (HR) is one of the most common parameters of assessing internal load in athletes easy and ecologically. Especially the use of percentage of the individual maximal HR (HRmax) is used to monitor internal load during training of different sports [4] and furthermore to define different intensity zones during the training process [5].

HR measurement with acquisition of the electrical signal transduced in bursts when R (R from the PQRS waveform curve of the ECG) peaks happen to show a high validity by using commercial HR chest belts compared to ECG [6, 7]. However, the use of chest belts during swimming is challenging. Especially male swimmers, with no swimsuit holding the chest belt in place have difficulties with the chest belt sliding down during free swimming and during turns. Recent sport watches use photoplethysmography (PPG) to obtain HR in different spots of the body. The accuracy of PPG devices has been shown to be affected by several environmental influences (i.e. sensor movement, pigmentation of the skin, tattoos etc.) [8–10]. The most common place for HR measurements with PPG is the wrist. Here several studies show different degrees of validity for different devices, sports, and intensities. (For more information the reader is referred to the meta-analysis by Giggins and Muggeridge [11]).

The Polar OH1 optical HR sensor (OH1) is a PPG device which can be used on different parts of the body. A recent study showed a high accuracy of the OH1 on the temple compared to ECG measurements during moderate to vigorous exercise on land [12]. The question arises if HR measurements on the temple with the OH1 also provide valid data during predominantly front crawl swimming with different intensities. Therefore, we investigated the validity of the Polar OH1 as well as a wrist worn PPG device Polar M600 smart watch (M600) during swimming by assessing the agreement with criterion measure Polar H10 HR monitor with a Pro Strap [13].

## Materials and methods

### Participants

Twenty-six well-trained competitive swimmers (13 women and 13 men; 18.1±2.3 yrs, 178.1±9.4 cm, 71.5±10.2 kg) of international and national performance levels participated voluntarily in the study (655.7±65.3 FINA Points).

The study was approved by the local Ethical committee of the Norwegian School of Sport Sciences (approval number 46–060218–200318) and the National Data Protection Agency for Research (approval number 58650) in accordance with the Declaration of Helsinki. The participants were given detailed verbal and written explanation of the purpose, procedures and risks associated with participation in the study. They completed a health history questionnaire including details on training activity levels, injuries and sickness prior to participation. Eligible participants or the legal guardian (for minors) then provided written informed consent prior to participation in the study. Prior to data collection, all participants were familiarized with the test procedures and equipment. Prior to the test day participants were instructed to abandon hard physical exercise over the last 48-hours.

### Experimental design

Participants performed a regular training session predominantly in front crawl including different swimming intensities. The session lasted between 70–85 minutes depending on the performance level of the swimmer. A detailed description of the whole training sessions performed by the participants with the main set according to the swimmer’s main distance being either 7x200-m (nine participants), 3x3x100 (nine participants), or a 4x8x50 (eight participants) step-test, can be found in [14]. Each bout began with the swimmers pushing off the pool wall. The different main sets where chosen to reflect the participants regular training sessions and based on whether the swimmer was predominantly a sprinter, middle-distance or a distance swimmer. The tests were performed in a 25-m indoor swimming pool with six lanes and water temperature of about 27°C. Participants were divided into three lanes with three to four swimmers per lane and highly experienced swim coaches with high knowledge of the participants training background followed each lane. They were responsible for organizing the lanes, following the participants with split times and giving continuous feedback on the pace. The tests took place between 05:00 and 08:00 PM.

### Data collection and analysis

During a prior testing session, the participants conducted a training session with the three different HR monitors that recorded HR continuously to familiarize themselves with the equipment. A Polar Pro chest strap was worn around the chest with Polar H10 HR sensor (H10) attached over the sternum (Polar Electro Oy, Kempele, Finland). During the test session, this was placed underneath a swimsuit. Male swimmers wore a suit covering their chest to avoid the chest strap from sliding down during the push-off from the wall. Polar OH1 optical HR sensor (OH1) (Polar Electro Oy, Kempele, Finland), was placed underneath the swimming cap at the temple through a customized headband made from an old chest strap. The placement of OH1 prevented hair from being present between the sensor and the temple. A sports watch with optical HR sensor, Polar M600 smart watch (M600) (Polar Electro Oy, Kempele, Finland), was placed on the left wrist of the participants.

HR data were collected from the entire training session, including periods of active and passive recovery, and stored in the internal memory of the devices. Polar Beat version 2.4.5 and 3.4.0 for Android (Polar Electro Oy, Kempele, Finland) was installed on each participant’s cellular phone for transferring HR data from H10 through Bluetooth. After completion of each test protocol, data was uploaded to Polar Flow (Polar Electro Oy, Kempele, Finland). After inspecting the data for possible abnormalities, they were exported to Microsoft Excel (Microsoft software, Microsoft Corporation, Redmond, WA, USA). HR from Polar H10 and Polar OH1 were live transmitted throughout the protocols through Bluetooth to a tablet (iPad 4, Apple, Inc.) where the participants were continuously monitored using Polar Team app version 1.3 (Polar Electro Oy, Kempele, Finland).

### Statistical analyses

Microsoft Excel and Statistica software package for Windows^®^ (version 7.1; StatSoft Inc., Tulsa, OK) were used for all statistical computations. A Shapiro-Wilk analysis was used to test for normal distribution of the data. A repeated-measured ANOVA with Bonferroni post-hoc analysis was used to compare HRmax, HRmean, and HRmin and for comparison of the percentage distribution of the training heart rate zones for the three different devices. P < 0.05 was considered significantly different. Effect size was calculated using eta squared between H10, and OH1 and M600 respectively.

To estimate any systematic bias, Bland-Altman analysis was used to test the agreement between H10 and OH1 and M600, respectively: bias (mean difference), standard deviation (SD) and upper and lower limits of agreement (LOA, defined as MD ± 1.96*SD) were calculated [15]. Intraclass correlation coefficients (ICCs) were calculated using H10, OH1, and M600 measurements for each swimmer [16]. This value indicates the validity of the OH1 and M600 measures vs. criterion (H10) (< 0.5: poor, 0.5–0.75: moderate, 0.75–0.9: good, > 0.9: excellent) [17].

## Results

In Table 1 the maximally achieved HR during the training session as well as the mean HR and the lowest HR is displayed. The maximal HR and mean HR obtained by the M600 was significantly lower (p < 0.05) than the HRmax and HRmean obtained by H10 and OH1. No difference in HRmax, HRmean and HR min between H10 and OH1 was evident. The ICC showed excellent agreements between H10 and OH1 and moderate to excellent agreements between H10 and M600 (Table 1).

**Table 1:** Descriptive statistics of the three different devices.

	*H10*	*OH1*	*M600*	*Effect size*		*ICC*	
				H10 - OH1	H10 - M600	H10 - OH1	H10 - M600
HRmax [bpm]	188.9±12.0	188.1±12.1	178.4±16.4 ^a^^,^^b^	.175	.493	0.987 *	0.570 *
HRmean [bpm]	140.6±11.0	139.5±11.8	131.1±11.2 ^a^^,^^b^	.207	.671	0.977 *	0.595 *
HRmin [bpm]	75.3±10.5	74.4±10.6	75.8±12.3	.069	.016	0.954 *	0.940 *

^a^: significantly different to H10 (p < 0.05),

^b^: significantly different to OH1 (p < 0.05),

*: significant correlation with H10 (p < 0.05)

Abbreviations: HR–heart rate; bpm–beats per minute; ICC–intra-class correlation coefficient

The validity of the OH1 sensors and the M600 was analyzed in different HR zones. Here the zones pre-defined by the Polar Flow app were used. Percent of the maximal HR measured by every device was used to calculate the time in the different intensity zones (<50%, 51–60%, 61–70%, 71–80%, 81–90%, 91–100% of HRmax, respectively). No significant difference for the percentage distribution of the HR obtained by H10 and OH1 was evident, while H10 and M600 showed three significant differences (Table 2). The ICC between H10 and OH1showed mostly good to excellent agreements with a moderate agreement for the 61–70% zone (Table 2). Agreements between H10 and the M600 were poor.

**Table 2:** Percentage distribution of the training heart rates with respect to the maximal heart rate measured by the particular device.

of HRmax	H10	*OH1*	*M600*	*Effect size*		*ICC*	
				H10 – OH1	H10 – M600	H10 – OH1	H10 – M600
91–100%	15.0±5.2	13.6±6.0	8.9±5.2 ^a^^,^^b^	.018	.591	0.845 *	0.299 *
81–90%	23.0±6.9	22.2±7.7	22.2±10.0	.088	.112	0.951 *	0.438 *
71–80%	24.7±8.1	25.3±9.4	28.5±9.2	.021	.136	0.903 *	0.358 *
61–70%	17.7±5.2	19.3±6.3	23.8±8.6 ^a^^,^^b^	.139	.353	0.720 *	0.209
51–60%	12.4±7.5	13.0±7.8	12.3±9.9	.037	.000	0.916 *	0.347 *
<50%	7.2±5.2	6.6±4.7	4.3±3.8 ^a^^,^^b^	.200	.269	0.963 *	0.366 *

^a^: significantly different to H10 (p < 0.05),

^b^: significantly different to OH1 (p < 0.05),

*: significant correlation with H10 (p < 0.05)

Abbreviations: HR–heart rate; ICC–intra-class correlation coefficient

**Fig 1:**
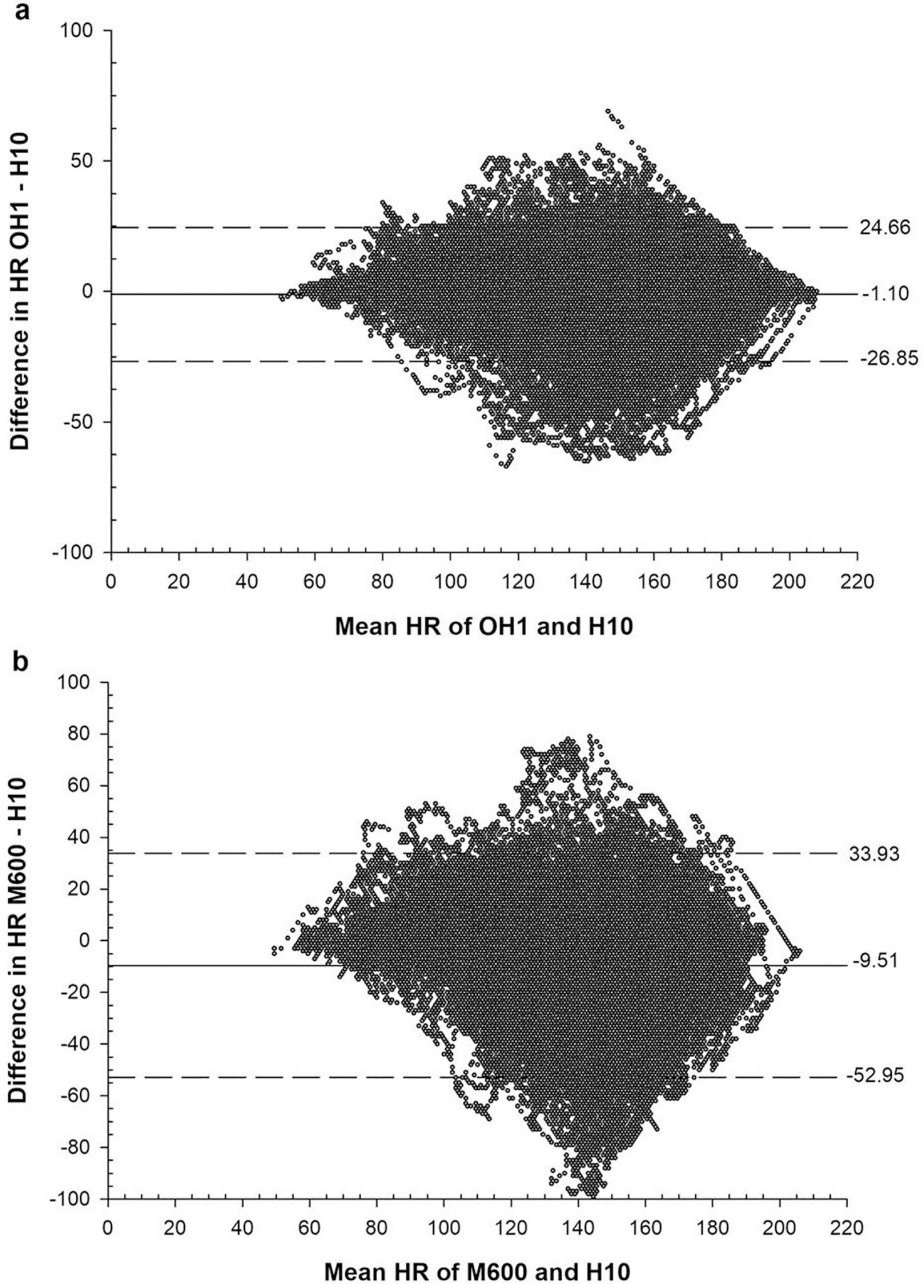
Bland-Altman-Plots of the H10, OH1 and M600 measurements, with bias (thick-black line) and with lower and higher values of agreement (dashed black lines). The sample size for the Bland-Altman-Plots are n = 112.400.

Fig 1 shows the Bland-Altman plots for the obtained HR of every swimmer with bias (thick- black line) and lower and higher values of agreement (dashed black lines).

**Fig 2:**
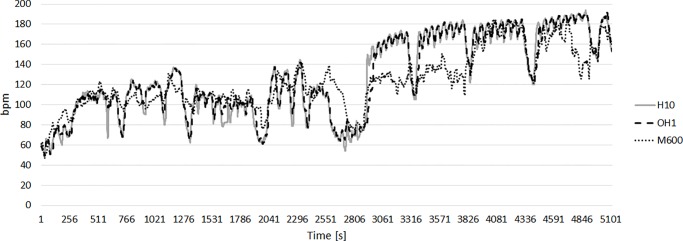
Example of the HR recordings of Polar H10 (solid grey line), OH1 (dashed black line), and M600 (small dashed black line) for one swimmer.

Fig 2 show one example of the HR recordings from the H10, OH1, and M600 for one swimmer. Whereas the signals for H10 and OH1 are mostly identical, the OH1 shows slightly delayed response in HR measurements at the beginning of short bouts of high intensity. The HR delay is more evident when recorded with M600.

## Discussion

The purpose of the present study was to compare the validity of a PPG heart rate monitor obtaining HR during swimming on the temple (OH1) to HR measured by a chest-belt (H10) and a PPG device worn on the wrist (M600). Our results show a high level of agreement between the OH1 and the chest belt (H10). The level of agreement between the M600 and the chest belt (H10) is low. We therefore suggest that the OH1 placed on the temple is a valid method of measuring HR during swimming.

The biggest challenge of measuring HR continuously during swimming in the past is the uncomfortable usage of a chest belt. The chest belt does not stay at the correct position during swimming and turns. Especially for male swimmers who are not using a full body suit. The PPG technique to measure HR at different sites of the body offered by numerous current sport watches might be an interesting alternative to the chest belt during swimming. PPG measurement is affected by several environmental influences (i.e. sensor movement, pigmentation of the skin, tattoos etc.) [8–10]. The most common place for HR measurements with PPG is the wrist. During dry-land exercises the validity of the PPG measurements during sport activities achieved by new watches is fairly high showing 80–90% agreement for running and cycling, respectively, with ECG measurements [18].

However, in a different environment like water it can be assumed that the fluid film between skin and watch might lead to a decrease in HR measurements. HRmax, HRmean and HRmin measured by the H10 and the OH1 were nearly identical (Table 1). The differences for HRmax and HRmean between H10 and M600 were statistically significant (p < 0.05) (Table 1).

Our results for the HR measurement on the wrist (M600) are in line with the findings for dry-land exercises [18]. The analysis of the Bland-Altmann plot indicates a wide range between the upper and lower limits of agreement for M600 (-53.0 to 33.9; Fig 1b). Reasons for these results might be due to motion artifacts during movements [9, 19, 20] and the fluid film between the watch and the skin. Another reason for the differences between the H10 and the OH1 compared to the M600 might be the slower “reaction time” of the measurements on the wrist. Especially in the beginning of high intensity intervals with a steep incline in HR the measurement by the M600 lags the measurement of the electrical activity obtained by the H10. Some authors suggest a lower peripheral resistance at the wrist which might diminish the changes in pulse pressure and therefore impede the detection of the blood pulse [18]. We might assume that the water leads to a lower skin temperature at the wrist compared to the temple and therefore blood circulation is further reduced.

This lower sensitivity of the PPG measurements found on the wrist was not evident for the PPG measurements by the OH1 at the temple. The upper and lower limits of agreement lay between -26.9 to 24.7 (see Fig 1a). There are some possible reasons which lead to an increase in validity of the PPG measurements at the temple with the OH1 compared to the PPG measurements at the wrist with the M600; i) the movements of the head during swimming are less pronounced compared to the arm movements reducing artefacts in the measurements; ii) the pulse pressure is higher at the temple and therefore the recognition of the blood pulse might be increased [18]; iii) the fluid film between the device and the skin is limited at the temple where the sensor is covered by the swim cap.

Monitoring training load has become an important part of the training process of athletes with the aim to understand and allocate training responses to a certain training content. Very often HR is used as an “easy to use” method to quantify training intensity. To prescribe and monitor training intensity mainly the percentage of HRmax is used [4]. The analysis of the intensity distribution in this study revealed no differences in the percentage distribution between the H10 and the OH1 with good to excellent ICCs. For the 61–70% zone the ICC showed only moderate agreements (Table 2). Reliability of the OH1 to assess training loads in endurance sports were high and lower for sports involving arm movements [21].

Besides the determination of statistical analyses and significances it is important to evaluate the practical relevance of the results. The training intensity distribution (TID) measured by the H10 revealed 42.4% of the training in the median intensity zones (61–80% HRmax), where it was 44.6% measured by the OH1 and 52.3% measured by the M600. For the high intensity zones (81–100%) the M600 measured only 31.1%, with 35.8% for the H10, and 38.0% for the OH1. This shows the practical problems which arise due to the slower “reaction time” leading to a worse discrimination between different intensity zones. In theory this might result in an underestimation of the intensities performed in training and potentially to an overreaching on the long term.
